# Towards a precise test for malaria diagnosis in the Brazilian Amazon: comparison among field microscopy, a rapid diagnostic test, nested PCR, and a computational expert system based on artificial neural networks

**DOI:** 10.1186/1475-2875-9-117

**Published:** 2010-05-06

**Authors:** Bruno B Andrade, Antonio Reis-Filho, Austeclino M Barros, Sebastião M Souza-Neto, Lucas L Nogueira, Kiyoshi F Fukutani, Erney P Camargo, Luís MA Camargo, Aldina Barral, Ângelo Duarte, Manoel Barral-Netto

**Affiliations:** 1 Centro de Pesquisas Gonçalo Moniz (FIOCRUZ), Bahia, Brazil; 2 Faculdade de Medicina da Bahia, Universidade Federal da Bahia (UFBA), Brazil; 3 Departamento de Ciência da Computação/Faculdade Ruy Barbosa, Salvador, Brazil; 4 Departamento de Ciências Biológicas, Universidade Estadual de Santa Cruz (UESC), Ilhéus, Bahia, Brazil; 5 Departamento de Microbiologia e Parasitologia, Universidade Federal de Santa Catarina, Florianópolis, Brazil; 6 Unidade Avançada de Pesquisa, Instituto de Ciências Biológicas V, Universidade de São Paulo (USP), Rondônia, Brazil; 7 Faculdade de Medicina, Faculdade São Lucas, Rondônia, Brazil; 8 Instituto de Investigação em Imunologia (iii), Instituto Nacional de Ciência e Tecnologia (INCT), São Paulo, Brazil; 9 Departamento de Tecnologia, Universidade Estadual de Feira de Santana (UEFS), Feira de Santana, Brazil

## Abstract

**Background:**

Accurate malaria diagnosis is mandatory for the treatment and management of severe cases. Moreover, individuals with asymptomatic malaria are not usually screened by health care facilities, which further complicates disease control efforts. The present study compared the performances of a malaria rapid diagnosis test (RDT), the thick blood smear method and nested PCR for the diagnosis of symptomatic malaria in the Brazilian Amazon. In addition, an innovative computational approach was tested for the diagnosis of asymptomatic malaria.

**Methods:**

The study was divided in two parts. For the first part, passive case detection was performed in 311 individuals with malaria-related symptoms from a recently urbanized community in the Brazilian Amazon. A cross-sectional investigation compared the diagnostic performance of the RDT Optimal-IT, nested PCR and light microscopy. The second part of the study involved active case detection of asymptomatic malaria in 380 individuals from riverine communities in Rondônia, Brazil. The performances of microscopy, nested PCR and an expert computational system based on artificial neural networks (MalDANN) using epidemiological data were compared.

**Results:**

Nested PCR was shown to be the gold standard for diagnosis of both symptomatic and asymptomatic malaria because it detected the major number of cases and presented the maximum specificity. Surprisingly, the RDT was superior to microscopy in the diagnosis of cases with low parasitaemia. Nevertheless, RDT could not discriminate the *Plasmodium *species in 12 cases of mixed infections (*Plasmodium vivax *+ *Plasmodium falciparum*). Moreover, the microscopy presented low performance in the detection of asymptomatic cases (61.25% of correct diagnoses). The MalDANN system using epidemiological data was worse that the light microscopy (56% of correct diagnoses). However, when information regarding plasma levels of interleukin-10 and interferon-gamma were inputted, the MalDANN performance sensibly increased (80% correct diagnoses).

**Conclusions:**

An RDT for malaria diagnosis may find a promising use in the Brazilian Amazon integrating a rational diagnostic approach. Despite the low performance of the MalDANN test using solely epidemiological data, an approach based on neural networks may be feasible in cases where simpler methods for discriminating individuals below and above threshold cytokine levels are available.

## Background

Despite global efforts, the malaria burden is increasing worldwide, with almost two million estimated deaths annually [1]. The lack of precise malaria diagnosis remains an important obstacle to the treatment adherence and effectiveness and clinical management of severe cases. Additionally, the invasiveness and expense of tests limit their utilization in asymptomatic individuals. Within the Brazilian Amazon, the microscopic detection and identification of *Plasmodium spp*. in Giemsa-stained blood smears from individuals presenting with malaria-like symptoms persists as the gold standard for the diagnosis of malaria and is mandatory to obtain access to the anti-parasitic treatment. Microscopic parasitological diagnosis requires continued personnel training and supervision of users in addition to a minimum laboratory structure, which is difficult to maintain in remote areas of the rainforest. Additionally, such a test is prone to large observer-related variation [2,3] and lacks sensitivity when performed by non-expert laboratory microscopists [4].

Other diagnostic methodologies have arisen to overcome the inefficient malaria diagnosis, such as PCR-based genetic tests. Nested PCR and real time PCR present higher sensitivity and specificity to malaria diagnosis compared to light microscopy [5,6]. Nevertheless, these molecular assays are costly and require even more laboratory support and personnel than microscopy, making it difficult to use routinely in the endemic areas. Rapid immunochromatographic tests (rapid diagnostic test, RDT) do not require laboratory support, are easily read and can reach a sensitivity similar to that commonly achieved by well-performed microscopy [7]. Nevertheless, most field evaluations of malaria RDTs were performed in countries with very high malaria endemicity [8,9], and validation studies in the Amazonian region are still scarce.

Within the Rondônia State in the Brazilian Amazon, the incidence of malaria and the occurrence of drug resistant cases are increasing [10]. In Buritis, a recently urbanized municipality, this situation is worsened by the lack of infrastructure of the health care system and the malaria control program. In addition, many other infectious diseases with similar clinical presentations, such as yellow fever, dengue and leptospirosis, are also common in this area, and the correct malaria diagnosis is of utmost importance to the adequate management of the patients. Certainly, one of the determining factors for morbidity and mortality is the delayed access to the health care. Moreover, the incidence of asymptomatic *Plasmodium *infection is very high in the Brazilian Amazon [11], further compounding the problem of malaria diagnosis. These individuals are not screened by the health care system, but they can transmit *Plasmodium *to uninfected *Anopheles *mosquitoes [12] and may represent important reservoirs. Therefore, the development of simple and noninvasive diagnostic tools is critical to hamper the spread of this infection.

Herein, the diagnostic effectiveness between an RDT (Optimal-IT), field microscopy and nested PCR was compared in individuals with malaria-related symptoms from an Amazonian region, which presents an increasing incidence of malaria [10]. Furthermore, a computational expert system based on artificial neural networks using epidemiological and clinical information was developed in an attempt to diagnose asymptomatic *Plasmodium *infection, and it was compared to field microscopy and nested PCR.

## Methods

### Ethics

This study was approved by the Ethical Committee of the São Lucas University, Rondônia, Brazil, for the human subject protocol. The clinical investigations were conducted according to the principles expressed in the Declaration of Helsinki. All participants or legal guardians gave written informed consent before patients entered the study.

### Participants and sampling

This study was performed in Rondônia State in the southwestern Brazilian Amazon. Within this region, the malaria transmission is unstable, with an increasing number of cases being detected annually from April to September [13]. Most malaria cases are caused by *P. vivax*. The prevalence of *P. falciparum *infection in the Brazilian Amazon is 23.7% [10], and the case detection of *Plasmodium malariae *is about 10% in Rondônia [14].

For the first part of the study, a cross-sectional investigation was performed between May 2006 and September 2007 in Buritis, Rondônia, Brazil (10°12'43" S; 63°49'44" W), a recently urbanized municipality with high prevalence of symptomatic malaria [10]. Passive malaria case detections were carried out in individuals who sought care at the diagnostic centers of the Brazilian National Foundation of Health (FUNASA), responsible for malaria control in the country. The purpose of this sampling method was to identify individuals with malaria presumptive symptoms. A total of 311 subjects enrolled in this part of the study.

To test the efficacy regarding the diagnosis of asymptomatic *Plasmodium *infection, riverine communities close to Demarcação, Rondônia, Brazil (8°10'04.12" S; 62°46'52.33" W), in which a high prevalence of asymptomatic *Plasmodium *infection has been reported [11], were studied. Active case detection was performed, which included home visits with interviews, clinical evaluations, and blood collection for nested PCR and cytokine measurements. Participants without any clinical evidence of malaria infection were assessed. All individuals who were living in the endemic area for more than six months and were asymptomatic were invited to be initially included in the study. Hence, a total of 380 individuals enrolled in the second part of the study. The baseline characteristics of the participants are illustrated in Table 1.

**Table 1:** Baseline characteristics of the subjects.

	Passive case detection	Active case detection
Number of participants	311	380
Age - years - median (range)	33.5 (4-65)	29.6 (10-72)
Male	188 (60.45%)	245 (64.47%)
Time of residence in the area - years - median (range)	6 (0.5-25)	14 (0.530)
Number of patients who reported previous malaria infections	303 (97.43%)	368 (96.84%)
Number of previous malaria infections reported - mean (range)	5 (0-12)	13.5 (9-45)

### The malaria diagnosis

The individuals were examined and interviewed by a trained physician. In the first part of the study, the thick blood smear and the Optimal-IT RDT (DiaMed China Ltd, Hong Kong, China) were run at the same time. The Optimal RDT was performed according to the manufacturer's instructions. For estimation of parasitaemia, experienced malaria field microscopists from the FUNASA malaria diagnostic center counted parasitaemia on slides using the thick film method. All the slides were stained using Giemsa pH 7.2. The results were reported as parasites/μL. In addition, 300 μL of total blood were collected in EDTA-treated tubes and stored for the nested PCR.

The molecular diagnosis of malaria infection was performed in all subjects using the nested PCR technique described previously [15]. To control for cross-contamination, one uninfected blood sample was included for every twelve samples processed. Fifteen percent of positive PCR samples were re-tested to confirm the amplification of plasmodial DNA. Part of the molecular assays was performed in the field laboratory facility (USP/ICB5, Monte Negro, Rondônia, Brazil). All tests were repeated and confirmed at the main laboratory at the Centro de Pesquisas Gonçalo Moniz, Bahia, Brazil. To certify that the individuals with a positive nested PCR test really had symptomless *Plasmodium *infections, they were followed for 30 days. Only the individuals who remained without malaria-related symptoms and positive nested PCR test after this period were classified as asymptomatic malaria cases.

### Expert System Based on Artificial Neural Networks

To identify asymptomatic *Plasmodium *infection, an expert system based on Artificial Neural Networks (ANN) [16] was developed using the epidemiological and clinical data. The software, called MalDANN (Malaria Diagnosis by Artificial Neural Networks), was built and validated using the data made available by a recent survey performed in malaria endemic areas in Rondônia State, Brazil, during 2006-2007, which was intended to study more deeply the causes that lead to asymptomatic malaria (unpublished observations). The MalDANN was developed in MATLAB 7.1 (MathWorks, Natik, MA, USA) using the Neural Network Toolbox for the construction of ANN.

The database provided by the survey contained 380 records with information from non-infected individuals (n = 178) and those with asymptomatic malaria (n = 202) (infected with *P. vivax *and/or *P. falciparum*) according to the nested PCR and clinical evaluation described above. The objective was to develop a helpful method for discriminating asymptomatic plasmodial infections from uninfected cases.

**Figure 1:**
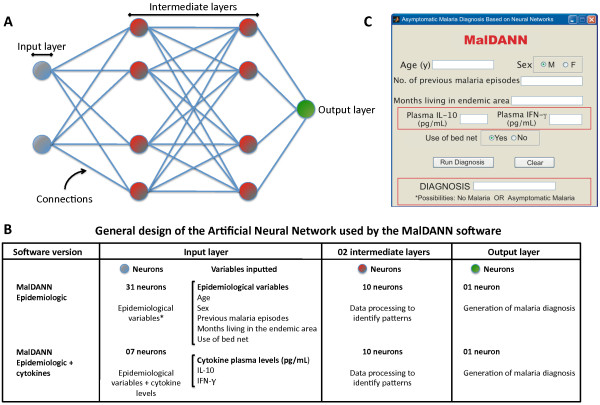
**General design of the Artificial Neural Network used by the MalDANN software**. (A) The neural network used by the MalDANN software was based on the Multilayer Perceptron, which consists of: (i) one input layer, where the standards and data are presented to the neural network; (ii) intermediate (or hidden) layers, where all the processing of the neural network is performed; and (iii) one output layer, in which the result of the network is presented to the observer. (B) Two software versions were created using different neural network structures to perform the diagnosis of asymptomatic *Plasmodium *infections. One version used epidemiological variables, and plasma levels of IL-10 and IFN-gamma were added to the epidemiological variables in the second version. (C) The intuitive interface of the MalDANN software was developed in order to facilitate the input of the data into the artificial network. * First, 31 epidemiological variables were added to the system for data mining. Of these, five variables presented very strong association with the asymptomatic malaria. The same five variables were added to the MalDANN version that used cytokine data.

The artificial neural network used in MalDANN was the Multilayer Perceptron because it is indicated for use in pattern recognition and provides the solution of problems not linearly separable [16,17] (Figure 1A). The network had one input layer (with seven neurons), two hidden layers (intermediate layers with four neurons each), one for each feature of the patient, and an output layer with only one neuron responsible for generating the diagnosis. The choice of activation functions of the layers of the neural network was made after a simulation of the activation functions provided by MATLAB. The best results were yielded by the function *tansig *in the input layer and hidden layers and the function *purelin *in the output layer. The network was trained using the back propagation technique in the Levenberg-Marquardt algorithm because it is very efficient when dealing with networks that have no more than a few hundreds of connections to be adjusted [18].

From the 380 records available in the database, a group of 300 records, approximately 80% of the total, were used for training, leaving 80 records for validation, approximately 20% of the total. In order to prevent a dominant class, a fact that could affect the training and evaluation of results, both the training data and the validation data had a balanced proportion of non-infected and asymptomatic infections.

### Plasma cytokine measurements

During the active search for subjects with asymptomatic *Plasmodium *infection, plasma levels of interleukin-10 (IL-10) and interferon-gamma (IFN-gamma) were measured using the Cytometric Bead Array - CBA^® ^(BD Biosciences Pharmingen, USA) according to the manufacturer's protocol, with all samples run in a single assay in the main laboratory at the Centro de Pesquisas Gonçalo Moniz, Bahia, Brazil. The flow cytometric assay was performed and analyzed by a single operator, and standard curves were derived from cytokine standards. The cytokine levels were used for other studies addressing pathogenic aspects of malaria in this region [15], and the information was used in the present study to check the impact of the cytokine balance on the prediction of asymptomatic malaria by the MalDANN software. Further, Receiver operator characteristic (ROC) curves were created with the values of each cytokine, and cut-off values presenting higher sensitivity and specificity were chosen to discriminate asymptomatic *Plasmodium *infections. The MalDANN software used this additional information together with clinical and epidemiological data to enhance the power of prediction of asymptomatic malaria cases.

### Statistical analysis

The overall performances of diagnostic methods were compared using Fisher's exact test (when two methods were compared) or a chi-square test (when three methods were compared). The results for the sensitivity, specificity, positive predictive values (PPV), and negative predictive values (NPV) obtained for each *Plasmodium *species were compared between the diagnostic methods using Fisher's exact test. Within all comparisons, differences in which p < 0.05 were considered statistically significant. The statistical analyses were made using the Graphpad Prism 5.0b (GraphPad Software, San Diego, CA, USA).

## Results

### Diagnosis of symptomatic *Plasmodium *infection

Of the 311 individuals presenting with malaria-related symptoms screened by FUNASA diagnostic centers, 188 (60.45%) were male, and the median age was 33.5 (Table 1). The individuals were living for less than seven years in the malaria endemic area (median of six years), and 303 (97.43%) of them reported at least one previous symptomatic *Plasmodium *infection (median number of previous malaria episodes: 5; interquartile interval: 0-12). Concerning the overall performance for the malaria diagnosis independently of the parasite species, the nested PCR resulted in the highest number of positive exams (173, 55.63% of the individuals), significantly superior to the RDT (154, 49.52%; p = 0.022, Fisher's exact test) and the field microscopy (141, 45.34%; p = 0.013, Fisher's exact test). As expected, the general positivity of the RDT was equivalent to the microscopy (p = 0.81; Fisher's exact test). Given that the nested PCR test presented higher positivity, it was considered as the gold standard to calculate the power of the two other tests. Therefore, for the diagnosis of symptomatic *Plasmodium sp*. infection, the RDT presented a sensitivity of 89.02% (95% CI: 83.38%-93.26%), a specificity of 100% (95% CI: 97.36%-100%), a positive predictive value (PPV) of 100% (95% CI: 97.36%-100%) and a negative predictive value (NPV) of 87.90% (95% CI: 81.75%-92.55%). Surprisingly, the light microscopy presented a lower sensitivity (81.50%; 95% CI: 74.9%-87.0%), an equivalent specificity (100%; 95% CI: 97.36%-100%) and PPV (100%; 95% CI: 97.42%-100%), and a lower NPV (81.18%; 95% CI: 74.48%-86.75%) than the RDT. Within the subjects evaluated in this study, no *P. malariae *case was detected. Under this circumstance, we decided to consider *P. non-falciparum *infection as *P. vivax *malaria cases for the RDT results.

Furthermore, the concordance of diagnosis in regard to the identification of the *Plasmodium *species was assessed (Table 2). The nested PCR detected a total of 107 individuals infected solely with *P. vivax *(61.84% of the positive cases), 53 individuals infected solely with *P. falciparum *(30.63% of the positive cases) and 13 cases of mixed infection (*P. vivax *+ *P. falciparum*; 7.51% of the positive cases). The light microscopy detected 84 cases of vivax malaria (23 cases fewer than nested PCR), 45 cases of falciparum malaria (eight cases fewer than nested PCR), and 12 mixed malaria cases (one fewer than nested PCR). The RDT detected 56 falciparum malaria cases and 98 vivax malaria cases. Importantly, the RDT used had limitations in the species identification because it intrinsically cannot discriminate mixed infections [19]. Hence, of the 12 cases of mixed infections detected by the nested PCR, the RDT discriminated as being eight cases of *P. falciparum *infection and four cases of vivax malaria. In a larger sample, this important issue can bring problems concerning the adequate management of the patients with mixed infections. Concerning the discrimination of *Plasmodium *species, both RDT and light microscopy presented similar performances (Table 3). Thus, compared to light field microscopy, the RDT was more powerful in the overall malaria diagnosis, presenting however an important undesired non-detection of mixed infections.

**Table 2:** Identification of symptomatic malaria cases: comparison among the field light microscopy, the Optimal-IT RDT and the nested PCR.

Microscopy	Optimal-IT^®^			Nested PCR			
		
	Pf	Pnf	Negative	Pf	Pv	Pf + Pv	Negative
Negative	3	10	157	9	23	0	138
*P. falciparum*	45	0	0	44	0	1	0
*P. vivax*	0	84	0	0	84	0	0
*P. vivax *+ *P. falciparum*	8	4	0	0	0	12	0

Total	56	98	157	53	107	13	138

Pf = *Plasmodium *falciparum, Pv = *Plasmodium *vivax, Pnf = Non falciparum *Plasmodium*.

**Table 3:** Performance of light microscopy and Optimal-IT in the discrimination of *Plasmodium *species.

*Plasmodium sp.*	Diagnostic test	Sensitivity	Specificity	PPV	NPV
		(95% CI)	(95% CI)	(95% CI)	(95% CI)
*P. vivax*	Microscopy	80%	100%	100%	88.8%
		(71.7-86.7)	(98.1-100)	(96.2-100)	(83.8-92.7)
	Optimal-IT	81.7%	100%	100%	89.7%
		(73.6-88.1)	(98.1-100)	(96.3-100)	(84.8-93.4)

*P. falciparum*	Microscopy	86.4%	100%	100%	96.5%
		(75.7-93.6)	(98.5-100)	(93.7-100)	(93.4-98.4)
	Optimal-IT	84.8%	100%	100%	96.1%
		(73.9-92.5)	(98.5-100)	(93.6-100)	(92.9-98.1)

The overall performance of Optimal-IT and light microscopy were compared to the nested PCR as the gold standard. No significant statistical difference was found between the tests for the diagnosis of both *P. vivax *and *P. falciparum*. CI, confidence interval; PPV, positive predictive value; NPV, negative predictive value.

The Table 4 presents the concordance of the tests' results according to the *Plasmodium *parasitaemia determined by light microscopy. Interestingly, a total of 32 patients were considered negative for malaria by the field microscopy specialized technicians from the FUNASA centers and were positive by nested PCR (23 vivax and nine falciparum malaria cases), while the RDT detected 13 cases (10 *P. vivax *and three *P. falciparum *infections). It was then possible that these patients had a parasitaemia below 100 parasites/μL because they were positive in two qualitative tests and negative by the parasite quantification using microscopy. On the other hand, the concordance of the results among the tests was similar when the patients presented with higher parasitaemia, except for the known absence of mixed infections detected by the RDT (Table 4). Further, the performance of RDT with microscopy in the infected subjects presenting with low parasitaemia, which was defined as <500 parasites/μL, was compared. The RDT was superior to microscopy concerning the diagnosis of *Plasmodium sp*. (76% vs. 59%, respectively), *P. falciparum *(75% vs. 63%, respectively) and *P. vivax *(76% vs. 58%, respectively) infections, with similar specificities and PPV (Table 5). Nevertheless, the microscopy had a higher NPV for *P. vivax *infections (88% vs. 75%, respectively). These findings indicate that for this endemic area, the RDT is superior to field light microscopy to identify individuals with low parasitaemia, albeit not detecting a few cases of mixed infections.

**Table 4:** Identification of symptomatic *Plasmodium *infection cases according to the parasitaemia.

Parasites/μL	Total	Microscopy				Optimal-IT^®^			Nested PCR			
		
		Pf	Pv	Pf+Pv	Negative	Pf	Pnf	Negative	Pf	Pv	Pf+Pv	Negative
Not detected	138	0	0	0	138	0	0	138	0	0	0	138
<100*	32	0	0	0	32	3	10	19	9	23	0	0
100 - 500	47	1	32	0	0	1	32	0	1	32	0	0
		5				5			5			
501 - 5,000	24	1	0	5	0	2	2	0	1	0	6	0
		9				2			8			
5,001 - 50,000	56	1	40	5	0	1	42	0	1	40	5	0
		1				4			1			
>50,000	14	0	12	2	0	2	12	0	0	12	2	0
Total	311	4	84	12	170	5	98	157	5	107	13	138
		5				6			3			

Pf = *Plasmodium *falciparum, Pv = *Plasmodium *vivax, Pnf = Non-falciparum *Plasmodium*.

* 32 subjects were negative for *Plasmodium *infection by field light microscopy. Nevertheless, nested PCR attested positive results (Optimal_IT identified 13 of them). Thus, these individuals probably present very low parasitaemia.

**Table 5:** Overall performance of microscopy and Optimal-IT in the discrimination of symptomatic malaria cases presenting with low parasitaemia.

Diagnostic method	*Plasmodium *species	Sensitivity	Specificity	PPV	NPV
Optimal-IT^®^	*Plasmodium non falciparum*	76%*	100%	100%	75%
	*P. falciparum*	75%†	100%	100%	97%
	*Plasmodium sp*	76%*	100%	100%	88%
					
Microscopy	*P. vivax*	58%	100%	100%	88%†
	*P. falciparum*	63%	100%	100%	96%
	*P. vivax + P. falciparum*	59%	100%	100%	81%

Values represent data from patients with <500 parasites/μL of blood determined by light microscopy. Nested PCR was considered the gold standard. PPV, positive predictive value; NPV, negative predictive value. The results for the sensitivity, specificity, PPV and NPV obtained for each *Plasmodium *species were compared between the diagnostic methods using Fisher's exact test. *p < 0.01; †p < 0.05.

### Diagnosis of asymptomatic *Plasmodium *infection

**Figure 2:**
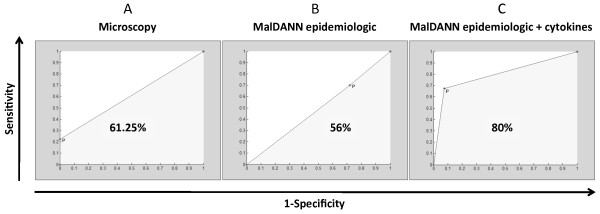
**Performance in discriminating asymptomatic *Plasmodium *infections**. In this investigation in an Amazonian riverine community, 380 apparently healthy individuals exhibiting no malaria-related symptoms were screened for *Plasmodium *infection by light field microscopy. Before the blood collection, the individuals were interviewed, and epidemiological data was obtained according to the methods. Whole blood and plasma samples were stored for nested PCR and cytokine measurements. The software MalDANN used epidemiological data only or in combination with the plasma levels of IFNgamma - and IL-10 to estimate the discrimination of the asymptomatic malaria cases. The overall performances of the field light microscopy (A) and the MalDANN (B and C) were compared using ROC curves, considering the nested PCR as the gold standard. The X-axes represent 1- specificity; the Y- axes represent sensitivity. Numbers inside the areas under the curves represent the percentages of correct diagnosis, which were statistically different using the chisquare-test (p = 0.002).

The next step was to assess the diagnosis of symptomless *Plasmodium*-infected individuals who are common in the Amazonian riverine communities [11] and may serve as infection source in endemic areas [12]. Previous studies have shown that individuals with asymptomatic malaria display distinct epidemiological characteristics from the symptomatic malaria cases [11]. In the present study, assessing another group of subjects from a riverine community, the field microscopy test correctly diagnosed only 61.25% of the samples (sensitivity: 22.5%; specificity: 100%; Figure 2A) when the nested PCR was considered the gold standard.

One artificial neural networks-based test (MalDANN) was developed, and fed with a data bank built during another study (Andrade *et al. *unpublished data). Initially, the information provided by the data bank included test results of several immunological parameters, socioeconomic, environmental, clinical and epidemiological data (Figure 1A-C). A multivariate analysis of such a databank was used to evaluate whether the variables of gender, age, number of previous malaria episodes, time of residence in the endemic area and the use of bed nets were associated with the asymptomatic cases (Figure 1B). In this analysis, all variables except for gender and the use of a bed net were associated to some extent with asymptomatic infection (Andrade *et al. *unpublished data). The ANN pre-processing of the same data displayed better prediction results when all of the cited variables were used, including gender and the use of a bed net.

These variables were then selected to be inputted in the MalDANN for the validation process in the present study. With this initial configuration, it was observed that the network did not reach an acceptable error rate (data not shown). It was noticed that among the training data were data from patients with very similar values but with different diagnoses. Hence, there was a need for greater number of neurons to process and better differentiate the pattern of input data. A new network was created using ten neurons in the hidden layers, keeping the other settings (Figure 1B). Thus the network achieved the error rate of 10-13 in 900 times, an error level considered acceptable for the proposed problem. After the software was designed, a very intuitive interface was developed to validate the ANN (Figure 1C).

Further, the diagnostic performances of the MalDANN and light microscopy were compared, considering nested PCR as the gold standard. Using exclusively the epidemiological data, the software diagnosed correctly only 56% of the samples (sensitivity: 70%; specificity: 28%), exhibiting lower performance than microscopic diagnosis due to increased number of false positive exams (72% vs. 0% of the samples tested, respectively; Figures 2A and 2B).

Additionally, the software was not able to discriminate the *Plasmodium *species. These data indicate that non-epidemiological variables may determine the occurrence of asymptomatic *Plasmodium *infection. To address whether the cytokine balance had a determining role in this process, plasma levels of IL-10 and IFN-gamma were measured, and the results were added into the neural network of the software. Strikingly, the software updated with the cytokine data correctly diagnosed asymptomatic malaria in 80% of the samples (sensitivity: 67.5%; specificity: 92.5%; Figure 2C) with a performance above field microscopy. Noteworthy, using the cytokine information (Figure 1B-C), the neural network reduced the false negative cases by 45% (Table 6). Interestingly, when added to the MalDANN system, other routine plasmatic biochemical laboratory exams, such as C reactive protein, fibrinogen, creatinine, haemoglobin, total bilirubin, direct bilirubin and indirect bilirubin, did not improved the diagnostic performance compared with the microscopy (MalDANN 60.4% vs. microscopy 61.25%; p = 0.6).

**Table 6:** Overall performance to discriminate asymptomatic malaria cases

Diagnostic method	Correct diagnosis (%)§**	True positive (%)**	True negative (%)***	False positive (%)***	False negative (%)**
Microscopy	61.25	22.5	100	0	77.5
MalDANN Epidemiologic	56	70	28	72	30
MalDANN Epidemiologic + cytokines	80	67.5	92.5	7.5	32.5

MalDANN: Malaria Diagnosis by Artificial Neural Networks. This diagnostic software was trained on the sample of 300 individuals actively screened for asymptomatic malaria and was validated in 80 other individuals, according to the methods. The diagnostic methods presented significantly different results estimated using the Chi-square test. §correct diagnosis involves both negative and positive correct exams. **p < 0.01; ***p < 0.0001.

## Discussion

The present study adds some relevant issues for the diagnosis of malaria in the Amazonian region. Firstly, only 55.63% of the individuals who sought care in a malaria diagnosis center presenting with malaria-related symptoms were diagnosed by the most sensitive diagnostic method applied in this study. The individuals with other diseases looked for exclusion of malaria before seeking care in a regular health clinic, possibly due to the high prevalence of malaria in the municipality. This custom can lead to a delay of the correct diagnosis and in severe cases, could compromise an adequate early management, directly impacting the prognosis and the cost of the health care.

The use of nested PCR as the gold standard was done when it was noted that it presented the highest sensitivity. Considering the principle of this molecular assay, in which small fragments of *Plasmodium *DNA can be detected, the results were not surprising. Nevertheless, it is worthy to evidence that until today the nested PCR is available only as a research tool, and the cost and technical complexity of this technique hamper the its use in quotidian screening and survey works. The rationale for choosing PCR as gold standard was the necessity of testing the diagnosis accuracy of the field microscopy and the RDT. In this study, 10.3% of the symptomatic individuals with a positive nested PCR (nine *P. falciparum *and 23 *P. vivax *cases) were negative by light field microscopy. Although these individuals probably had low parasitaemia, they were symptomatic and did not receive anti-malarial treatment because of the negative microscopy exam. This finding reinforces that the field microscopists from this area need continued refinement, and investments are necessary to improve the quality of the malaria screening. It also indicates that the large demand for microscopic tests in this area, including those from patients with other infections, could contribute to the reduced quality of the tests. Recent evidence indicates that there is a large inter-rater reliability of the parasite counts for the malaria diagnosis [3]. The thin film method is not feasible at a parasitaemia below 500 parasites per microlitre, while the thick film method gives slightly better inter- rater agreements [3]. Moreover, it is well known that most routine malaria microscopists require constant retraining, and that their ability to detect a high proportion of malaria cases is suspect [20].

In addition, the RDT presented higher effectiveness in the identification of malaria cases with low parasitaemia than the light microscopic test. Many other studies worldwide have indicated diverse findings [5,21,22]. This result reinforces the idea that assays for rapid diagnosis have the potential to enhance diagnostic capabilities in those instances in which skilled microscopy is not readily available [23]. In order to identify individuals with low parasitaemia neglected by the light microscopy screening in this endemic area, the use of a RDT is advisable in symptomatic individuals who presented a negative thick blood smear exam. This method should be tested in field conditions but it will likely expand the detection of infected individuals and may favour the early clinical intervention and adequate case management. In the sample of 311 individuals, this approach would have resulted in the use of 32 RDT tests, with a minor impact of the health care cost compared to the possible outcomes resulting from a delayed diagnosis.

Optimal-IT, the RDT used in the present study, cannot discriminate mixed infections. The discrimination between *P. vivax *and *P. falciparum *infections is critical because the drug therapies and the treatment durations are different. Actually, there are other RDT that can discriminate mixed infections [24], and these should be validated in this endemic area because the occurrence of mixed infections found here was 4.2%. The choice of using the Optimal-IT in this study was made because FUNASA was validating its use in the field during the study period. This work took advantage of this occasion and decided to compare the power of this RDT with other diagnostic tools.

The asymptomatic *Plasmodium *infection is a major problem in many regions worldwide [25,26], including the Brazilian Amazon [11]. Symptomless individuals probably develop clinical immunity to *Plasmodium *parasites after repeated infections [27], which lead to modifications on the host physiology that minimize the intensity of the symptoms, maintaining a very low parasitaemia for long periods [28]. While under this occult infection, these individuals have no reason to seek care in the malaria diagnosis centers. On the other hand, the quotidian primary care activities do not include active detection of asymptomatic malaria. Consequently, these symptomless individuals remain parasitaemic and can serve as a parasite source for uninfected mosquitoes [12], which in turn favours the spread of the infection. Knowledge ofthe prevalence of asymptomatic malaria cases in certain regions could assist in the implementation of control strategies, which may include treatment of asymptomatic *Plasmodium*-infected individuals.

Asymptomatic individuals frequently refuse to give blood for tests, which hampers the detection of symptomless plasmodial infections. Additionally, the routinely used thick blood smear exam displays a low performance in individuals with low parasite burdens, as is the case in asymptomatic *Plasmodium *infection [29]. Herein, a pilot investigation was performed addressing whether a computational system could discriminate asymptomatic malaria cases. For this purpose, major epidemiological determinants of asymptomatic malaria, such as the age, time of residence in the endemic area, number of previous malaria infections, gender and use of bed nets, were used. The technique of ANN was chosen because it offers good robustness against noise and typically works very well when no previous knowledge is available in order to facilitate the classification [18]. Thus, the network can be trained to recognize the pattern of the disease to be diagnosed from the medical database used. With a very intuitive interface that could be used by primary care professionals in the endemic areas, the software used an expert system based on neural networks [16]. The disappointing results obtained by the exclusive use of epidemiological data indicate that other complex factors may be more influential for the development of the asymptomatic *Plasmodium *infection. This idea was confirmed when the software performance significantly improved after the addition of information regarding IL10 - and IFN-gamma plasma measurements. In addition, other routine biochemical laboratory exams did not improved the MalDANN performance. These cytokines were chosen in the light of evidences that individually or as ratios they are associated with the malaria severity [15,30]. Besides the known epidemiological factors, the genetic background and/or the common occurrence of co-infections within the population may play a fundamental role on the occurrence of asymptomatic malaria.

## Conclusion

This study shows that the chosen RDT (Optimal-IT) performed superiorly in discriminating symptomatic malaria cases with low parasitaemia than field microscopy, although it did not discriminate mixed infections. An RDT for malaria diagnosis may find a promising use in the Brazilian Amazon integrating a rational diagnostic approach. Despite the low performance of the MalDANN test using solely epidemiological data, an approach basedon neural networks may be feasible in cases where simpler methods for discriminating individuals below and above threshold cytokine levels are available.

## Competing interests

The authors declare that they have no competing interests.

## Authors' contributions

Wrote the paper: BBA; Performed data analysis: BBA and ARF; Performed the field study and clinical examinations: BBA, SMSN and LMAC; Performed molecular experiments: LLN, KFF and BBA; Designed and validated the Expert Based System Networks for malaria diagnosis: AMB and AD; Participated in the design of the study and helped with the manuscript: LMAC, EC and AB; Coordinated the study and helped to draft the manuscript: MBN. All authors have read and approved the final version of the manuscript.

## Authors' information

BBA, ARF, SMSN and LLN received fellowships from the Brazilian National Research Council (CNPq). EC, AB and MBN are senior investigators from CNPq. This work was supported by FINEP (010409605)/FNDCT-CT Amazônia. The funders had no role in the study design, data collection and analysis, decision to publish or preparation of the manuscript.
